# Shared decision making and behavioral impairment: a national study among children with special health care needs

**DOI:** 10.1186/1471-2431-12-153

**Published:** 2012-09-21

**Authors:** Alexander G Fiks, Stephanie Mayne, A Russell Localio, Chris Feudtner, Evaline A Alessandrini, James P Guevara

**Affiliations:** 1 The Pediatric Research Consortium (PeRC), The Children's Hospital of Philadelphia, 34th Street and Civic Center Boulevard, Philadelphia, PA, 19104, USA; 2 Center for Biomedical Informatics (CBMI), The Children's Hospital of Philadelphia, 34th Street and Civic Center Boulevard, Philadelphia, PA, 19104, USA; 3 Center for Pediatric Clinical Effectiveness, The Children's Hospital of Philadelphia, 34th Street and Civic Center Boulevard, Philadelphia, PA, 19104, USA; 4 PolicyLab, The Children’s Hospital of Philadelphia, 34th Street and Civic Center Boulevard, Philadelphia, PA, 19104, Pennsylvania; 5 Department of Pediatrics, Perelman School of Medicine at the University of Pennsylvania, 3620 Hamilton Walk, Philadelphia, PA, 19104, USA; 6 Department of Biostatistics and Epidemiology, Perelman School of Medicine at the University of Pennsylvania, 3620 Hamilton Walk, Philadelphia, PA, 19104, USA; 7 The James M. Anderson Center for Health Systems Excellence, Cincinnati Children’s Hospital Medical Center, 3333 Burnet Avenue, Cincinnati, OH, 45229, USA

**Keywords:** Children with Special Health Care Needs, Communication, Decision-Making

## Abstract

**Background:**

The Institute of Medicine has prioritized shared decision making (SDM), yet little is known about the impact of SDM over time on behavioral outcomes for children. This study examined the longitudinal association of SDM with behavioral impairment among children with special health care needs (CSHCN).

**Method:**

CSHCN aged 5-17 years in the 2002-2006 Medical Expenditure Panel Survey were followed for 2 years. The validated Columbia Impairment Scale measured impairment. SDM was measured with 7 items addressing the 4 components of SDM. The main exposures were (1) the mean level of SDM across the 2 study years and (2) the change in SDM over the 2 years. Using linear regression, we measured the association of SDM and behavioral impairment.

**Results:**

Among 2,454 subjects representing 10.2 million CSHCN, SDM increased among 37% of the population, decreased among 36% and remained unchanged among 27%. For CSHCN impaired at baseline, the change in SDM was significant with each 1-point increase in SDM over time associated with a 2-point decrease in impairment (95% CI: 0.5, 3.4), whereas the mean level of SDM was not associated with impairment. In contrast, among those below the impairment threshold, the mean level of SDM was significant with each one point increase in the mean level of SDM associated with a 1.1-point decrease in impairment (0.4, 1.7), but the change was not associated with impairment.

**Conclusion:**

Although the change in SDM may be more important for children with behavioral impairment and the mean level over time for those below the impairment threshold, results suggest that both the change in SDM and the mean level may impact behavioral health for CSHCN.

## Background

Shared decision making (SDM) is defined as the active participation of both clinicians and families in treatment decisions, the exchange of information, discussion of preferences, and a joint determination of the treatment plan [1]. Given benefits of SDM in increasing families’ knowledge, decreasing uncertainty, and pairing families with treatments they find most acceptable [2], the Institute of Medicine (IOM) recently stressed the importance of research assessing the comparative effectiveness of SDM in pediatrics [3] and the 2010 Patient Protection and Affordable Care Act supported the implementation of SDM in clinical settings [4]. Despite the prioritization of research on SDM, little work has investigated the association of SDM with children’s health.

SDM is particularly useful when families must balance the risks and benefits of more than one evidence-based treatment. This process therefore may be especially helpful in the management of behavioral problems since families choose between behavior therapies and medical treatments, often informed by strongly held personal and cultural values. Concerns such as cost, accessibility, stigma, effectiveness, and side effects have been shown to shape these decisions [5]. By addressing families’ preferences, SDM explicitly incorporates values into the decision making process. As a result, beliefs that could undermine treatment acceptability, engagement and adherence may be discussed prior to the selection of therapeutic options and potentially lead to a better match between families and treatments.

Children with special health care needs (CSHCN), those who have or are at increased risk of a chronic physical, developmental, behavioral, or emotional condition and who require health and related services beyond that required by children generally [6], are an ideal population for the study of the impact of SDM on behavioral health. Approximately 10 to 12 million United States (US) children have special health care needs [7], up to 30% of CSHCN have a behavioral or emotional disorder [8,9], and behavior problems are often more severe among CSHCN than others [10,11]. Furthermore, even for children without a diagnosed behavioral or mental health condition, the presence of a special health care need often increases the need for mental health services for both affected children and their families [9].

To address these often multi-faceted problems, collaboration between clinicians and families has become a priority for these children. Ensuring “that families partner in decision making at all levels and are satisfied with the services they receive” is one of the Maternal and Child Health Bureau’s 6 core outcomes for CSHCN [12]. Still, little attention has focused specifically on the association of SDM with children’s behavioral health and how patterns of SDM over time may impact behavioral outcomes. To address this knowledge gap and provide guidance for pediatric clinicians who routinely treat behavioral problems, we conducted a longitudinal study of SDM and behavioral health among a national sample of US CSHCN. We hypothesized that both higher mean levels of SDM over time as well as increasing SDM would be associated with improved behavioral health.

## Methods

### Study design and data source

We conducted a longitudinal analysis of the Medical Expenditure Panel Survey (MEPS), administered annually by the Agency for Healthcare Research and Quality (AHRQ) and previously used to study CSHCN [13,14]. Between 12,810 and 14,828 households were sampled annually from the US population, and all children were followed for 2 years [15]. In MEPS, the person from each household who was most knowledgeable about the health of its members provided information on health status, insurance, and utilization. Interviews were supplemented by surveys from medical providers, health insurers, and employers.

### Study sample

The study sample included all children ≥5 and ≤17 years in MEPS panels 7 to 10 (2002- 2006). Younger children were excluded because behavioral impairment is not assessed in MEPS. From this sample, CSHCN were identified using the validated CSHCN Screener [16,17]. Children were excluded if they had no usual source of care (SDM does not apply in this context), their household did not respond to any of the items used to create the SDM measure, or they lacked a value for impairment. Response rates for completion of all survey rounds for the years considered ranged from 58.3% to 64.7% [15]. We were able to generalize results to the US population of CSHCN by applying sampling weights that reflect the number of people in the US represented by each respondent.

### Outcome measure

The primary outcome was behavioral impairment in the domains of interpersonal relationships, psychopathology, functioning at school, and use of leisure time as assessed during each of the 2 study years by the validated, parent-reported, 13-item Columbia Impairment Scale (CIS), which has previously been used with CSHCN [18,19]. Each item is scaled from 0 (no problem) to 4 (a very big problem) and total scores ≥15 indicate impairment. Even small differences in mean score may be clinically meaningful. Differences between 1 and 3 points have been found between groups of children with and without physical abuse [20] and with and without major depression [21].

### Independent variables

SDM was the primary independent variable. We determined families’ participation in SDM during each study year by calculating the mean item score of responses to 7 separate MEPS items that address distinct aspects of SDM. These items correspond to the four components of SDM in the most widely accepted definition (Table 1) [1]. After excluding children without a response to any of the 7 SDM items, for each item, 7-15% of remaining children lacked a response. Multiple imputation with 10 data sets was then used to address missing data on these items in a manner designed to avoid bias and produce correct confidence intervals [22]. In order to examine the impact of both the mean level of SDM over the 2 study years and the change in SDM from year 1 to year 2 on behavioral impairment, we considered 2 aspects of SDM in the analysis. First, we calculated the mean SDM for each subject across the 2 study years ((year 1 mean SDM + year 2 mean SDM)/2). Second, to address the change in SDM, a pattern of increasing, decreasing, or unchanged SDM, we calculated the difference between each subject’s SDM score in each year and their mean SDM score over the 2 study years. This approach effectively partitioned the change across the 2 study years and centered all of the change at zero, allowing a longitudinal analysis to be conducted as was appropriate for the data structure.

**Table 1:** Items Included in the Shared Decision Making Score

**Shared Decision Making Items from the Medical Expenditure Panel Survey**	**Corresponding Component(s) of the Definition of Shared Decision Making (See Below).**	**Unweighted distribution of scores (n, %)**^**1**^			
		**1 (Never)**	**2 (Sometimes)**	**3 (Usually)**	**4 (Always)**
If there were a choice between treatments, how often would your medical provider ask you to help make the decision?	1, 4	186 (8%)	277 (11%)	591 (24%)	1400 (57%)
Thinking about the types of medical, traditional and alternative treatments you are happy with, how often does your medical provider show respect for these treatments?	3	62 (3%)	191 (8%)	584 (24%)	1617 (65%)
In the last 12 months, how often did your child’s doctors or other health providers listen carefully to you?	2,3	19 (1%)	143 (6%)	541 (22%)	1751 (71%)
In the last 12 months, how often did your child’s doctors or other health providers explain things in a way that you could understand?	2, 3	19 (1%)	125 (5%)	460 (19%)	1850 (75%)
In the last 12 months, how often did your child’s doctors or other health providers show respect for what you had to say?	3,4	20 (1%)	124 (5%)	477 (19%)	1833 (75%)
In the last 12 months, how often did your child’s doctors or other health providers spend enough time with you?	2	53 (2%)	166 (7%)	559 (23%)	1676 (68%)
		1 (No)			4 (Yes)
Does a medical person at your usual source of care present and explain all options to you?	2	144 (6%)			2310 (94%)
**Components of Shared Decision Making**					
(1) Both the doctor and the patient are involved in the treatment decision-making process;					
(2) Both share information with each other;					
(3) Both take steps to participate in the decision-making process by expressing treatment preferences;					
(4) Both the doctor and the patient ree on the treatment to implement					

^1^Missing data (7-15% for each item) was imputed using multiple imputation.

### Covariates and effect modification

We considered as covariates clinical and demographic variables that might impact the relationship between SDM and behavioral impairment (Table 2). These covariates consisted of demographic characteristics including the child’s age (5-12 versus 13-17 years), gender, race (White, Black, other) and Hispanic ethnicity, region of residence (Northeast, Midwest, South, West), parental education (no high school diploma, high school diploma, bachelor’s degree, graduate level degree, or other degree), and household income (poor (<100% of the applicable poverty line), near poor (100 to <125%), low (125 to <200%), middle (200 to <400%), high (≥400%)) as well as any private health insurance (versus other insurance or none). To ensure that findings were not confounded by child health, we adjusted for general health status based on the overall score (low (<15), medium (15 to <20), and high (≥20)) from 5 Likert-scaled items derived from the parent-reported Child Health Questionnaire, General Health Subscale [23]. Specifically, we controlled for whether health status improved, remained unchanged, or declined between years 1 and 2 of the study. To control for the impact of specific behavioral treatments, we also adjusted for whether children received any psychotropic medications (includes stimulants, antidepressants, antipsychotics, anticonvulsants, as well as other psychotropic medications such as alpha agonists) or any mental health services (visits to a psychiatrist, psychologist, or other mental health professional) in only the first year, only the second year, both study years or never.

**Table 2:** **Comparing Characteristics of Children with Special Health Care Needs (CSHCN), Age 5-17 Years, by Shared Decision Making Pattern**^**1**^

**Variable**	**Included**	**Excluded**	***P*value comparing Included vs. excluded**^**2**^
Number of children in sample	2454	174	
Number of children represented in population	10.2 million	641,000	
Percent represented	94%	6%	
**Demographic Characteristics**	%	%	
Age (Years)			
5-12	70.0	64.1	0.2
13-17	30.0	35.9	
Female	44.0	44.3	0.9
Race			
White	78.4	69.6	0.009
Black	16.1	18.5	
Other	5.5	11.9	
Hispanic	12.5	16.9	0.1
Region			
Northeast	19.1	8.1	0.02
Midwest	22.8	22.7	
South	37.8	49.2	
West	20.3	20.0	
Parental Education			
No Degree	10.3	17.8	0.1
High School Complete	46.4	48.2	
Bachelor's Degree	16.7	12.8	
Graduate Level Degree	11.9	11.6	
Other Degree	14.7	9.6	
Poverty			
Poor	18.4	22.2	0.007
Near Poor	5.9	8.9	
Low Income	15.0	24.9	
Middle Income	31.5	27.5	
High Income	29.2	16.5	
Insurance Coverage			
Any private	65.4	52.9	0.01
Other	34.6	47.1	
**Clinical Characteristics**	%	%	
General Health Status^3^			
Increasing	20.4	20.1	0.9
Unchanged	59.8	60.4	
Decreasing	19.8	19.5	
Diagnosed with ADHD	19.8	8.3	0.001
**Variable**	**Included**	**Excluded**	***P*value comparing Included vs. excluded**^**2**^
Diagnosed with Asthma	20.1	19.3	0.8
Any psychotropic medication use^4^			
None	70.7	85.6	0.01
Year 1 only	2.9	2.8	
Year 2 only	6.1	3.9	
Both years	20.3	6.7	
Any mental health services use^5^			
None	76.5	68.8	<0.001
Year 1 only	6.1	20.1	
Year 2 only	6.8	5.8	
Both years	10.6	5.3	

^1^Children excluded from the study lacked a usual source of care, had no response to any of the items used to create the SDM measure, or lacked a response to items from the Columbia Impairment Scale used to assess behavioral impairment.

^2^*P* values calculated by chi-square tests with robust variance estimates accounting for the weighted, clustered, and stratified longitudinal MEPS survey design.

^3^General Health Status determined using the overall score from 5 Likert-scaled items (recoded so that a score of 5 indicates the best health and a score of 1 the worst health) derived from the Child Health Questionnaire, General Health Subscale (child seems less healthy than other children, child has never been seriously ill, child usually catches whatever is going around, expect child will have a healthy life, respondent worries more than is usual about child’s health). For each year, the overall score was categorized as (low (<15), medium (15 to <20), or high (≥20)).

^4^ Psychotropic medications considered included stimulants, antidepressants, antipsychotics, anticonvulsants, as well as other psychotropic medications such as alpha agonists.

^5^ Mental health services include visits to a psychiatrist, psychologist, or other mental health professional.

Because our initial analyses demonstrated that the association of SDM with behavioral impairment differed with the baseline level of impairment, we evaluated our primary outcome separately in CSHCN who were impaired at baseline and in those who were unimpaired. Although the study sample size was too small to test effect modification formally based on diagnosis or treatment receipt, we conducted separate secondary analyses including clinically relevant subpopulations: children diagnosed with asthma, children with attention-deficit/hyperactivity disorder (ADHD), those taking psychotropic medication, and those utilizing mental health services.

### Statistical analysis

#### Characteristics of the study sample

We initially described the weighted proportion of CSHCN in MEPS included versus excluded from the study and compared the characteristics of CSHCN with each pattern of SDM using chi-square tests. We then plotted each subject’s SDM score in year 1 versus year 2 to observe differences over time, and calculated Pearson correlation coefficients and p-values. Following guidance from AHRQ, all statistical analyses accounted for the weighted, clustered, and stratified MEPS survey design, an approach that provides conservative estimates that account for repeated measures over time [24]. In all analyses, P values of <0.05 were considered significant.

#### Assessing the impact of SDM on behavioral impairment

To assess the association between SDM and CIS score over time, linear models with robust variance estimates that reflect the complex survey design were created with the change in CIS score as the outcome and with the mean level of SDM score across the two years as well as the change in SDM as the 2 independent variables. The use of robust variance estimates as well as change scores limited the impact of the skewed distribution of SDM on confidence bounds and significance levels in our analyses. These models were developed separately for CSHCN who were impaired at baseline and those who were unimpaired. In each analysis, we constructed models with and without covariates. Since the inclusion of covariates did not alter the association of SDM with behavioral health, covariates were dropped from the final models presented in the Results section. Analyses used Stata 10 and 11 (College Station, Texas). The CHOP Institutional Review Board determined this study, which involved only de-identified, publically available data, to be exempt from IRB oversight.

## Results

### Study sample

Based on the survey methodology, the study sample of 2,454 represented a population of 10.2 million US CSHCN. This sample included 94% of the weighted population of US CSHCN (Table 2). Compared to included children those excluded were significantly more likely to be of Black or other race, to be from the South, to have higher levels of poverty, and no private health insurance. Excluded children were also less likely to be diagnosed with ADHD, less likely to be taking psychotropic medication, and less likely to be utilizing mental health services in both years.

### Extent of behavioral impairment and patterns of shared decision making

At baseline, the mean CIS score among all CSHCN was 10.6 out of 52 and 34.2% of CSHCN had impaired behavior (score ≥15 represents impairment). Baseline CIS scores were higher among children diagnosed with ADHD (15.9 versus 9.3, p < 0.001) and lower among those with asthma (8.9 versus 11.0, p < 0.001) compared to other CSHCN. Similarly, CIS scores were higher for those on psychotropic medication versus none (16.7 versus 8.7, p < .001) and receiving mental health services versus none (18.0 versus 9.1, p < .0001).

**Figure 1:**
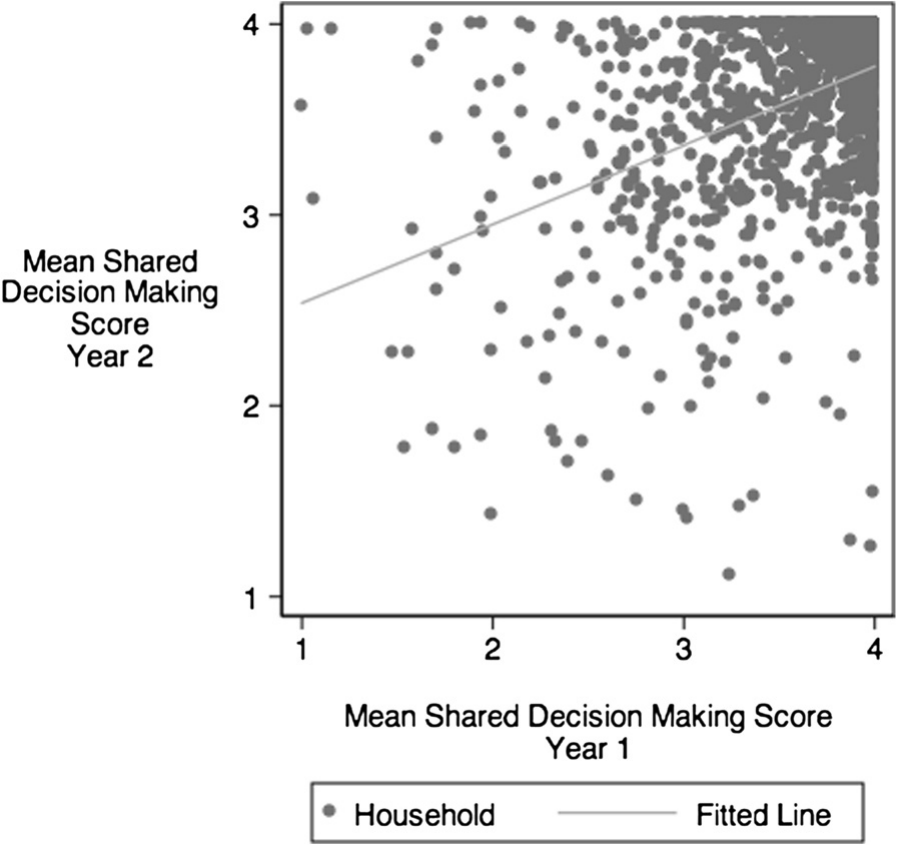
**Shared decision making scores in year 1 and year 2: overall high correlation of scores (r = 0.8, p < .001), with substantial individual variability.** Fitted line reflects the correlation between year 1 and year 2.

At baseline, the mean SDM score among all CSHCN was 3.6 out of 4. The distribution of responses to the SDM items is shown in Table 1. Figure 1 plots the mean SDM score for each subject in year 2 by the mean score in year 1. While the SDM score in year 1 was strongly associated with SDM score in year 2 (r = 0.7, p < 0.001), 37% of families reported an increase in SDM, 36% reported a decrease, and only 27% remained unchanged. Among those whose SDM score increased, 88% increased by <1.0 (out of 4.0 possible), 11% increased by 1.0 to <2.0, and 1% increased ≥2.0. Among those whose SDM score decreased, 90% decreased by <1.0, 9% decreased by 1.0 to <2.0, and 1% decreased by ≥2.0.

### Association of shared decision making with behavioral impairment

**Figure 2:**
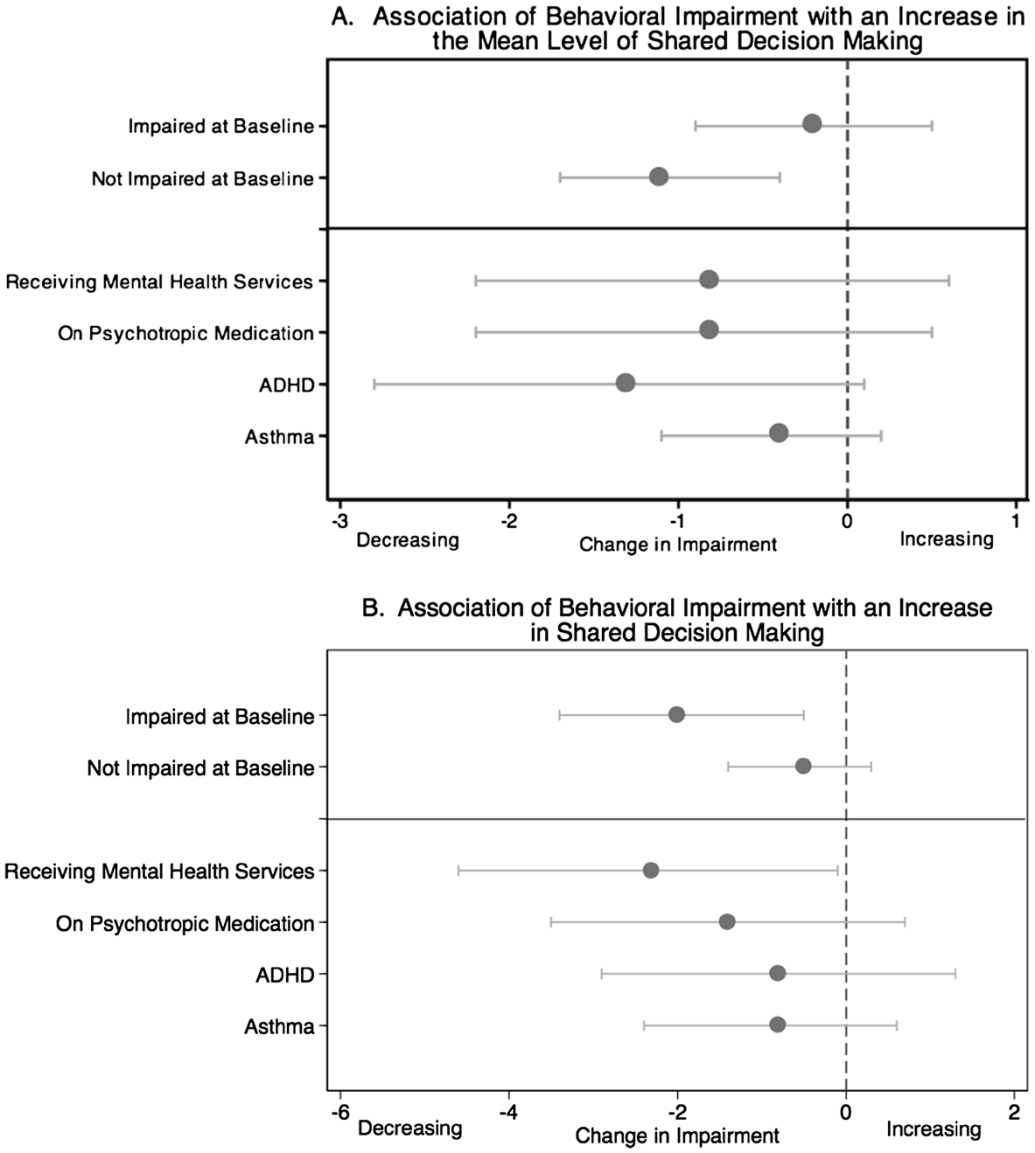
**Different groups of CSHCN are affected more by either the mean level of SDM or by an increase in SDM.** Plot A shows the decrease in impairment associated with each 1-point increase in the mean level of SDM. Plot B shows the decrease in impairment associated with each 1-point increase in SDM over time, a measure of the change in SDM.

The impact of SDM on behavioral impairment differed among CSHCN who were impaired versus unimpaired at baseline (Figure 2). Among CSHCN who were not impaired, behavioral impairment scores decreased by 1.1 points (0.4, 1.7) with each one point increase in the mean level of SDM (Figure 2A). In this group, the change in SDM over time was not significantly associated with impairment (Figure 2B). In contrast, for CSHCN impaired at baseline, the change in SDM was significant with each 1-point increase in SDM over time associated with a 2-point decrease in impairment (95% CI: 0.5, 3.4). However, the mean level of SDM was not significantly associated with impairment.

In secondary analyses of subgroups of CSHCN, results were only significant for those receiving mental health services, the subgroup with the highest impairment (Figure 2). Consistent with our main results, the change in SDM, but not the mean level of SDM, was significant with each 1-point increase in SDM over time associated with decreases in impairment of 2.3 points (95% CI: 0.1, 4.6). Trends were similar for other subgroups.

## Discussion

In this study, the first national, longitudinal research project examining the association of SDM with behavioral impairment in children, we hypothesized that both higher sustained levels and increasing patterns of SDM would be associated with decreased impairment among CSHCN. While consistent with our hypothesis, study results showed different patterns of SDM may be most beneficial for children with versus without high levels of impairment at baseline. We found that higher mean levels of SDM were associated with improvements among children below the threshold for behavioral impairment, but only an increase in SDM was associated with significantly improved behavioral health among those with impairment. Although prospective study is warranted to more thoroughly detail how SDM impacts behavioral health, these novel results suggest that increases in SDM may be needed to achieve the best outcomes for children with behavioral impairment, while sustained SDM over time may be more effective to help CSHCN with behavior problems that adversely affect families but fall below the impairment threshold.

A possible explanation for these findings may be the distinct types of decisions that pediatric clinicians and families share when children have varying levels of impairment. When children are severely impaired, SDM is likely to focus on starting new medical or behavioral treatments that may result in substantial improvements in behavior. Increasing SDM in this context may maximize the likelihood that these treatments are optimized with input from the family in order to best limit impairment. Sustained SDM, as reflected in the mean, might be less important in this context than increasing SDM specifically when major decisions are reached. In contrast, when behavior problems fall below the impairment threshold, clinicians may instead focus on incremental interventions that make many small improvements over time. In this context, sustained SDM in which families and clinicians jointly address minor difficulties over a period of more than one year may help reduce impairment. Since our findings are novel, further study is needed to better understand and confirm these relationships.

While not directly addressing SDM, the few pediatric interventions designed to improve behavioral health by either fostering communication or the medical home, a broader term including care coordination as well as SDM, have had mixed results. A trial of communication skills training for urban pediatric clinicians led by a psychiatrist and drawing from techniques of family-centered care, family and cognitive behavioral therapy, and family engagement, decreased parent-rated impairment among minority, but not white children with behavior problems [25]. A study of the 2007 National Survey for Children’s Health found that children with ADHD cared for in a medical home were less likely to have difficulties participating in activities or making friends [26]. In a rural area, a program to enhance comprehensive, coordinated care for CSHCN with nurse practitioner home visits, goal setting, and follow-up resulted in improved functioning for the family, but not the child [27].

In the context of mixed findings from these interventions, the benefits of SDM in our results justify prospective study examining the impact of interventions to foster SDM on childhood behavioral health. Extensive research primarily targeting adults has been devoted to developing decision aids, standardized and validated tools specifically created to foster SDM by helping families consider the risks and benefits of specific treatments in the context of their personal values [2]. Decision aid use results in improved decision making, but not consistently improved health outcomes [2]. Unlike decision aids which focus on optimizing individual decisions, the items used in this study assess SDM in the broader context of care which may explain the difference in our findings. Since prior research indicates that improved communication between families at home and their pediatricians is strongly associated with SDM [28] but that SDM occurs inconsistently in clinical encounters with impaired children [29], two types of interventions may be needed: decisions aids to foster SDM within encounters and office-based systems such as expanded telephone hours or patient portals to foster ongoing collaboration and SDM. Office-based staff may also provide outreach to enhance the ongoing shared decision making process.

The longitudinal design of this study allowed us to investigate how patterns of SDM change over time, an area that has received little attention in prior research. We found that 36% of households reported increasing, 37% decreasing, and 27% unchanged SDM. Among possible explanations for these shifts, SDM may increase as families gain experience with different treatment modalities and are better able to participate or may increase or decrease when families switch clinicians and a new partnership develops. Child health status as well as families’ outlook and level of stress, factors known to influence pediatric palliative care decision making [30], may also impact families’ and clinicians’ willingness and ability to participate in SDM.

While this study is the first to use a nationally representative sample to assess the impact of SDM on children’s behavioral health over time, it has several limitations. Although we considered 7 items with face validity based on their correspondence with the definition of SDM [1], additional items might have allowed us to further refine our measure and better assess variability in SDM. SDM has been conceptualized as existing between the extremes of paternalistic decision making by the doctor alone and informed decision making by the patient or family alone [1]. However, our study measure limited us to assessing only extent of family involvement in decision making, not who ultimately made decisions. In addition, we relied on household report as opposed to direct observation of SDM. We could not verify how options were presented. Families also might have been more likely to report increased SDM when their children became less impaired, an association that might have biased our results. Although we adjusted for child health status, a similar construct to impairment, and found no impact on results, this might not have fully accounted for the impact of impairment on reported SDM. Furthermore, while our data set provided a national perspective, we conducted an observational study that used a single measure of behavioral health. Trials are needed to definitively assess how SDM impacts behavior using multiple measures of the process and outcomes. Given limits in our sample size that prevented formal tests of effect modification, these studies should also distinguish the impact of SDM in the general population of CSHCN versus among those with primarily behavioral or physical conditions.

## Conclusions

We found that SDM was associated with improved behavioral health for US CSHCN. However, different patterns of SDM were associated with improved behavioral health for children with higher versus lower levels of impairment. These results suggest that increasing SDM may be needed to achieve the best outcomes for children with the greatest behavioral impairment, while sustained SDM over time may be more effective to help CSHCN with behavior problems that fall below the impairment threshold. Prospective research is needed to evaluate the impact of strategies to both augment and sustain SDM for this population.

## Abbreviations

SDM: Shared Decision Making; IOM: Institute of Medicine; CSHCN: Children with Special Health Care Needs; US: United States; MEPS: Medical Expenditure Panel Survey; AHRQ: Agency for Healthcare Research and Quality; CIS: Columbia Impairment Scale; ADHD: Attention-Deficit/Hyperactivity Disorder; CHOP: Children’s Hospital of Philadelphia; IRB: Institutional Review Board.

## Competing interests

The authors declare that they have no competing interests.

## Author’s contributions

AF conceived of the study, participated in the design and in planning the analysis, and drafted the manuscript. RL, EA, JG, and CF participated in the design of the study and in planning the analysis. SM performed the statistical analysis and assisted in drafting the manuscript. All authors read and approved the final manuscript.

## Pre-publication history

The pre-publication history for this paper can be accessed here:

http://www.biomedcentral.com/1471-2431/12/153/prepub
